# Gut microbiota profile in systemic sclerosis patients with and without clinical evidence of gastrointestinal involvement

**DOI:** 10.1038/s41598-017-14889-6

**Published:** 2017-11-01

**Authors:** Vania Patrone, Edoardo Puglisi, Marco Cardinali, Tobias S. Schnitzler, Silvia Svegliati, Antonella Festa, Armando Gabrielli, Lorenzo Morelli

**Affiliations:** 1 Dipartimento di Scienze e Tecnologie Alimentari per una filiera agroalimentare sostenibile (DiSTAS), Facoltà di Scienze Agrarie, Alimentari ed Ambientali, Università Cattolica del Sacro Cuore, Via Emilia Parmense 84, 29122 Piacenza, Italy; 2 Clinica Medica, Dipartimento di Scienze Cliniche e Molecolari, Università Politecnica delle Marche, Via Tronto 10/A, 60126 Ancona, Italy

## Abstract

Recent evidence suggests that there is a link between the gut microbial community and immune-mediated disorders. Systemic sclerosis (SSc) is an autoimmune disease characterized by immunonological abnormalities, vascular lesions, and extensive fibrosis. Since the gastrointestinal tract is one of the organs most involved, the goal of this study was to explore the composition of the intestinal microbiota in SSc patients with (SSc/GI+) and without gastrointestinal involvement (SSc/GI-) in comparison to healthy controls (HC). The fecal bacterial composition was investigated by Illumina sequencing of 16 S rRNA gene amplicons. The fecal microbiota of SSc/GI+ subjects was characterized by higher levels of *Lactobacillus*, *Eubacterium* and *Acinetobacter* compared with healthy controls, and lower proportions of *Roseburia*, *Clostridium*, and *Ruminococcus*. The gut microbiota of SSc/GI- subjects was more similar to the microbiota of HC than to that of SSc/GI+ subjects albeit *Streptococcus salivarius* was over-represented in SSc/GI- fecal samples compared with both SSc/GI+ subjects and controls. Our study reveals microbial signatures of dysbiosis in the gut microbiota of SSc patients that are associated with clinical evidence of gastrointestinal disease. Further studies are needed to elucidate the potential role of these perturbations in the onset and progression of systemic sclerosis, and gastrointestinal involvement in particular.

## Introduction

Systemic sclerosis (scleroderma, SSc) is an autoimmune, clinically heterogeneous and poorly understood disorder of the connective tissue, characterized by vascular lesions, immunological abnormalities, and fibrosis of skin and internal organs^1,2^. Systemic sclerosis is a severe disease, a major cause of disability with a high morbidity and mortality directly related to the extension of fibrosis and microvasculature alterations^3^. Several reports have shown that the gastrointestinal tract is frequently involved, with 80% of SSc patients having esophageal dysfunction and 40–70% having involvement of the stomach, small and large intestine^3,4^. In longstanding disease upper gastrointestinal involvement occurs in nearly all patients. The spectrum of symptoms range from bloating, to heartburn, dysphagia, malabsorption and severe weight loss, all of which have a great impact on the quality of life. Standard pharmacological treatments provide limited benefit and does not modify the course of the disease. Much effort has been lately focused on earlier detection of gastrointestinal involvement and effective intervention to slow its progression^5,6^.

The term gut microbiota describes the entire intestinal microbial communities dominated by mainly anaerobic bacteria and other micro-organisms such as Archaea, Eukarya, viruses and fungi^7^. Recent studies have established the important role played by the gut microbiome in modulating vital functions of the healthy host and in autoimmunity inducing either stimulation^8–10^ or protection^11,12^ or in some cases no effect^13^. Furthermore, the host plays an important role in shaping the microbiota^14^. In light of the above considerations it is common knowledge that the gut microbiota may be involved in the pathogenesis of immune-mediated disorders including type I diabetes, multiple sclerosis, rheumatoid arthritis, inflammatory bowel diseases^15^.

Recently, Volkmann and colleagues studied 17 SSc patients, and found that the patients, as compared to healthy, unrelated controls, harbored a distinct colonic microbiota in cecum and sigmoid lavage samples obtained during colonoscopy, with decreased levels of commensal bacteria and increased levels of pathobiont bacteria^16^.

With the present study, conducted in a geographically and ethnically distinct cohort of SSc patients we wanted i) to confirm that dysbiosis is a pathological feature of systemic sclerosis as also suggested by a further study^17^; ii) to verify whether the findings described by Volkmann and Colleagues are specific of SSc or, instead, related to the specific characteristics of the population they studied; iii) to assess whether the presence and absence of gastrointestinal involvement in systemic sclerosis is associated with a distinct microbiota composition. To this end, we compared the fecal microbiota of two groups of SSc patients to that of healthy controls. One group was composed by SSc patients with gastrointestinal involvement (SSc/GI+), while the second group (SSc/GI-) did not have clinical evidence of gastrointestinal disease.

## Results

### Characteristics of the study subjects

Demographic, clinical, and immunological features of the 18 enrolled patients are summarized in Table 1. SSc/GI+ patients had a mean age of 55.3 (36 to 79) years and a mean disease duration (defined as the time from the onset of the first non-Raynaud’s phenomenon clinical manifestation) of 10.7 years. All patients were female, and Caucasian. Two patients (22%) had limited cutaneous SSc and 7 patients (78%) had diffuse cutaneous SSc. The majority of the patients, 4 (44%), were anti-topoisomerase-I-positive; 2 (22%) of the patients were anti-centromere-positive. SSc/GI- patients had a mean age of 57.4 (34 to 78) years and a mean disease duration (calculated from the detection of immunological or capillaroscopic alterations) of 6.7 years. One patient (11%) was male and 8 patients (89%) were female, all Caucasian. Four patients (44%) were anti-centromere-positive, while one (11%) had anti-topoisomerase-I antibodies. The control group had a mean age of 54.8 (26 to 78) years. Eight subjects were female, and 1 male. The patients and controls had similar physical activity and dietary habits. The analysis of the food frequency questionnaires showed according to chi-squared tests no significant differences in the consumption patterns of rice, wheat, vegetables, fish or meat between the study groups (data not shown).

**Table 1:** Characteristics of the study subjects. Significant differences according to ANOVA are highlighted by p values.

	SSc/GI-*	Healthy Controls	SSc/GI+**	p***
**Age, mean (range) years**	57.4 (34–78)	54.8 (26–78)	55.3 (36–79)	0.75
**Mean disease duration**	6.7 (range 2–13) years		10.7 (range 2–22) years	0.3
**Male**	1	1	0	0.65
**Female**	8	8	9	
**Limited cutaneous SSc**	9 (100%)	—	2 (22%)	0.0002
**Diffuse cutaneous SSc**	0	—	7 (78%)	0.0002
**Antinuclear Antibody Positive**	8 (88%)	—	9 (100%)	1
**Anti -topoisomerase I antibody positive**	1 (11%)	—	4 (44%)	0.29
**Anti centromere antibody positive**	4 (44%)	—	2 (22%)	0.37
**Diffuse abdominal pain**	0	—	7 (78%)	0.002
**Bloating**	0	—	7 (78%)	0.002
**Pyrosis**	0	—	6 (67%)	0.009
**Epigastric pain**	0	—	4 (45%)	0.08
**Gastric fullness**	0	—	3 (33%)	0.2
**Diarrhea**	0	—	3 (33%)	0.2
**Constipation**	0	—	2 (22%)	0.47
**Active smokers**	0	2 (22%)	0	
**BMI** ^**¶**^	24.11 ± 2.1	25.8 ± 2.3	19.9 ± 2.36	0.0002
**FVC** ^**§**^	90 ± 10.9	97 ± 15	80.77 ± 17.2	0.019
**DLCO** ^**‡**^	86.6 ± 10.9	90 ± 17	59.22 ± 30.26	0.0004
**m RSS**	8.88 ± 4.28	0	15.11 ± 6.88	0.0004
**Presence of SIBO**	0	—	3 (33%)	0.2
**UCLA SCTC GIT 2.0 questionnaire, mean** ± **SD** ^**Þ**^				
**Distension/bloating score**	0	0	1 ± 0.4	0.002
**Reflux score**	0	0	0.6 ± 0.3	0.07
**Fecal soilage score**	0	0	0	0.96
**Diarrhea score**	0	0	0.9 ± 0.2	0.002
**Emotional well-being score**	0	0	0.6 ± 0.5	0.003
**Social functioning score**	0	0	0.4 ± 0.4	0.076
**Constipation score**	0	0	0.4 ± 0.4	0.076
**N of subjects tested/studied**				
**UCLA SCTC GIT 2.0 questionnaire**	9/9	0/9	9/9	
**Glucose and lactulose breath test**	0/9	0	3/9	
**Chest TC scan**	0/9	0	6/9	
**Modified barium swallow**	0/9	0	9/9	
**13C octanoid acid breath test**	0	0	4/4	
**Albumin**	4.03 ± 0.6	4.2 ± 0.3	3.6 ± 0.82	0.03
**Ferritin**	85.77 ± 22.8	150 ± 15	14.66 ± 13.61	0.001
**Cholesterol**	191.22 ± 63.4	200 ± 20	175.33 ± 40	0.03
**PT** ^**#**^	98.88 ± 11.8	105 ± 2	91.22 ± 21	0.02
**C Reative protein**	0.34 ± 0.25	0.2 ± 0.1	0.43 ± 0.27	0.32
**ESR (mm/h)** ^**¥**^	17.22 ± 16.1	12 ± 3	17.77 ± 9.19	0.59
**Use of PPI** ^**ð**^	0	0	0	
**Use of immunosuppressive agents**	0	0	0	
**Use of prokinetics drugs**	0	0	0	

*SSC/GI- = systemic sclerosis patients without gastrointestinal involvement; **SSC/GI+ = systemic sclerosis patients with gastrointestinal involvement;

***p = comparison of SSC/GI+ with SSC/GI-; − = absent.

^¶^BMI = Body mass index.

^§^FVC = Forced vital capacity.

^‡^DLCO = diffusing capacity of the lung for carbon monoxide.

m RSS = modified Rodnan skin score.

SIBO = small intestine bacterial overgrowth.

^Þ^multi-tier scales of UCLA SCTC GIT 2.0 questionnaire are listed below.

^#^PT = Prothrombin time;

^ð^PPI = proton-pump inhibitors;

^¥^ESR = Erythrocyte sedimentation rate.

### Illumina 16S analyses of fecal microbiota

Assembly and demultiplexing of the Illumina paired-end sequences resulted in a total of 2,199,437 sequences, progressively reduced to 1,093,347 (elimination of homopolymers and sequences <380 bp), 1,021,194 (elimination of sequences aligning out of the targeted V3-V4 16S rRNA region), 966,344 (elimination of chimeras) and finally 963,487 (elimination of sequences not classified as bacterial after alignment against the SILVA database)^18^.

Differences in number of sequences per sample are prone to generate errors in diversity estimates^19,20^: to avoid these errors, sequences per samples were downscaled to 8,679, which was the number of sequences of the lowest populated sample. The Good’s coverage index after this rarefaction step was still very high (96.7% ± 2%), thus showing that most of the bacterial diversity in the samples was still captured. Furthermore, specific tests on Good’s coverage were carried out before and after rarefaction, in order to see if the operation led to any significant loss in the captured diversity. Prior to rarefaction, group SSc/GI+ had 80,429 ± 19,914 (average ± standard deviation), group SSc/GI- 15,395 ± 1747, HC 11,546 ± 2,709; the Good’s coverage indexes were stable before and after rarefaction for all three groups, passing from 99.5% to 99.0% for SSc/GI+, from 94.9 to 94.2% for SSc/GI- and from 95.0 to 94.2% for HC. This comparison thus shows that the rarefaction to 8,679 sequence per sample didn’t result in a loss of captured diversity, especially for the SSc/GI+ group which was much more affected by the rarefaction than the other two.

**Figure 1:**
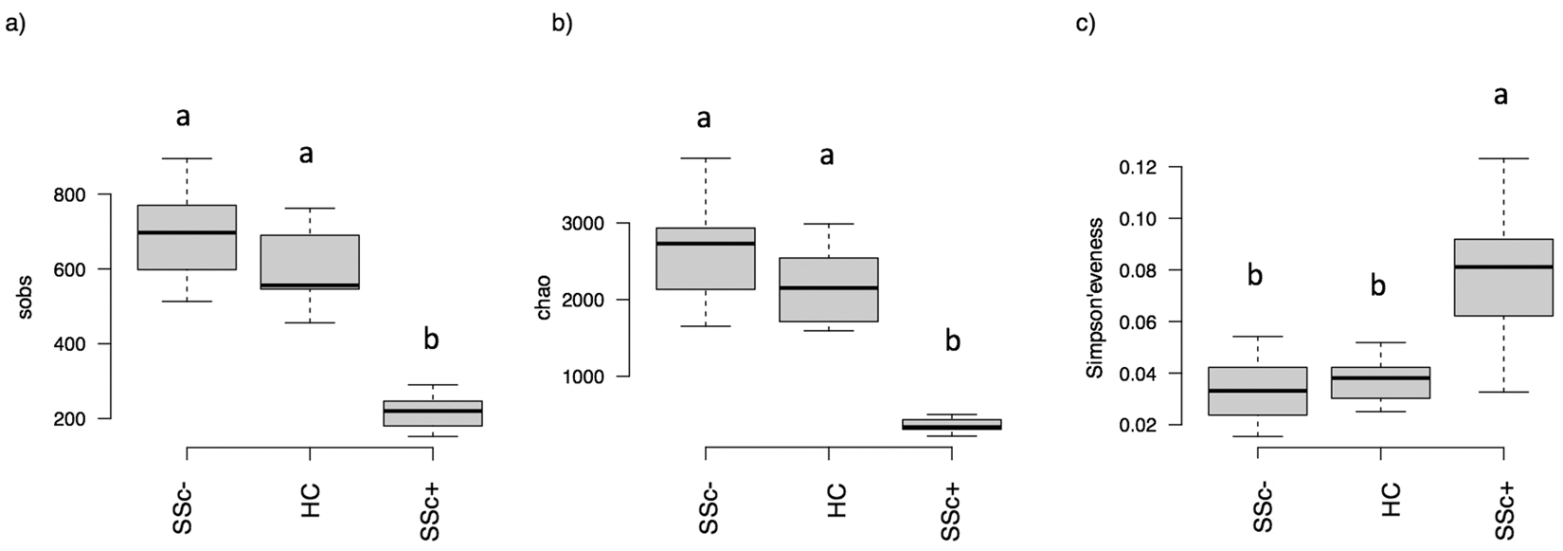
Gut microbiota diversity in the fecal samples of the experimental groups of subjects. Boxplots show (**a**) the total number of OTUs, (**b**) Chao index and (**c**) Simpson’s eveness indexes in patients with established SSc and gastrointestinal symptoms (SSc/GI+), patients with SSc without gastrointestinal symptoms (SSc/GI-), and healthy controls (HC), respectively. Significant differences are highlighted by different minor letters according to ANOVA and Tukey’s honestly significant difference (HSD) test for comparison of means (P < 0.05).

Analyses were carried out on OTUs (Operational Taxonomic Units) at 97%, which are reliable estimates of bacterial species, without any sub-filtering for rare OTUs. α-diversity indexes were calculated in order to assess the diversity within the gut bacterial community in each patient and control (Fig. 1). Results showed no differences in terms of total measured OTUs (observed S) between healthy subjects and SSc/GI- patients; on the contrary, SSc/GI+ patients were separately grouped, with a strong reduction of total OTUs, dropping from an average of 709 and 604 in the SSc/GI- and healthy group respectively down to 216 total OTUs in the SSc/GI+ subjects (Fig. 1a). Accordingly, also the Chao index (Fig. 1b) did not show differences between the first two groups, while being almost ten times lower (357 as compared to 2767 and 2228) in the SSc/GI+ subjects. On the contrary, the latter group had significantly higher values for the Simpson evenness index, a measure of the relative abundance of OTUs, albeit with a higher standard deviation as compared to the other two α-diversity indexes (Fig. 1c).

**Figure 2:**
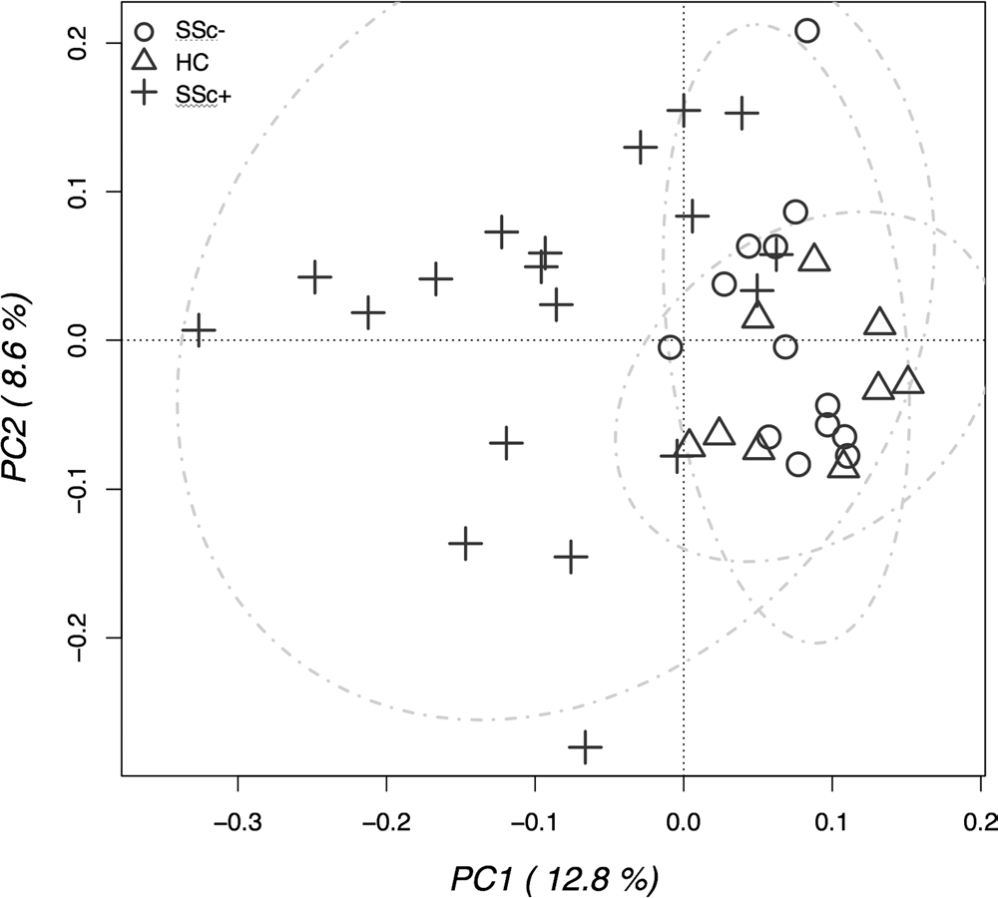
Principal component analysis (PCA) of fecal microbiota compositions in the experimental groups of subjects. PCA was performed based on abundance of classified OTUs with frequency >99.9% in patients with established SSc and gastrointestinal symptoms (SSc/GI+), patients with SSc without gastrointestinal symptoms (SSc/GI-), and healthy controls (HC), respectively. The percentages on each axis indicate the variation in the samples. Individuals are labeled according to the three groups studied.

**Figure 3:**
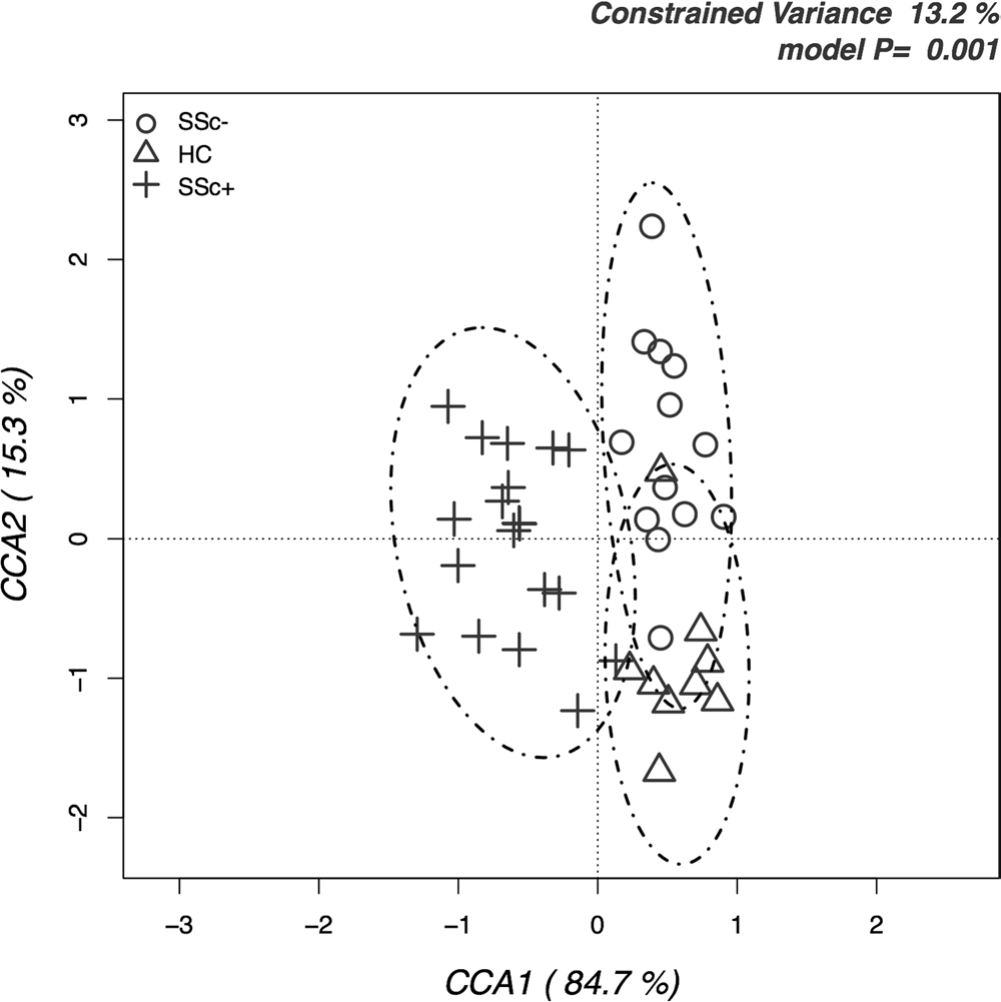
Hypothesis-driven canonical correspondence analysis (CCA) of fecal microbiota compositions in the experimental groups of subjects. CCA was performed based on abundance of classified OTUs with frequency >99.9% in patients with established SSc and gastrointestinal symptoms (SSc/GI+), patients with SSc without gastrointestinal symptoms (SSc/GI-), and healthy controls (HC), respectively. The plot shows that the disease status is a significant source of variability in bacterial communities, explaining 13.2% of the variance in fecal microbiota. The percentages on each axis indicate the variation in the samples. Individuals are labeled according to the three groups studied.

Distances between the microbiome of all analyzed subjects were evaluated by multivariate β-diversity analyses, firstly by principal component analysis (PCA, Fig. 2) and then by canonical correspondence analysis (CCA, Fig. 3). PCA results were in agreement with α-diversity indexes, since they showed a full overlapping of healthy individuals and SSc/GI- individuals; the SSc/GI+ group showed instead a higher variability between individuals, with most of them clustering apart from the other two groups. These patterns in the bacterial ecology of the individuals were further explored by CCA, a constrained method that allows testing if a classification variable (in this case the health state) is significantly affecting the relative distribution of OTUs across individuals. Results showed indeed a significant *P* value of 0.001, although with 17.9% of variance explained (Fig. 3). SSc/GI+ individuals were indeed separated from the other two groups along the first major axis, while the healthy and SSc/GI- groups were also separated along the second axis. This latter outcome suggests that an in-depth analysis of microbiota has the potential for discrimination of patients without gastrointestinal symptoms.

**Figure 4:**
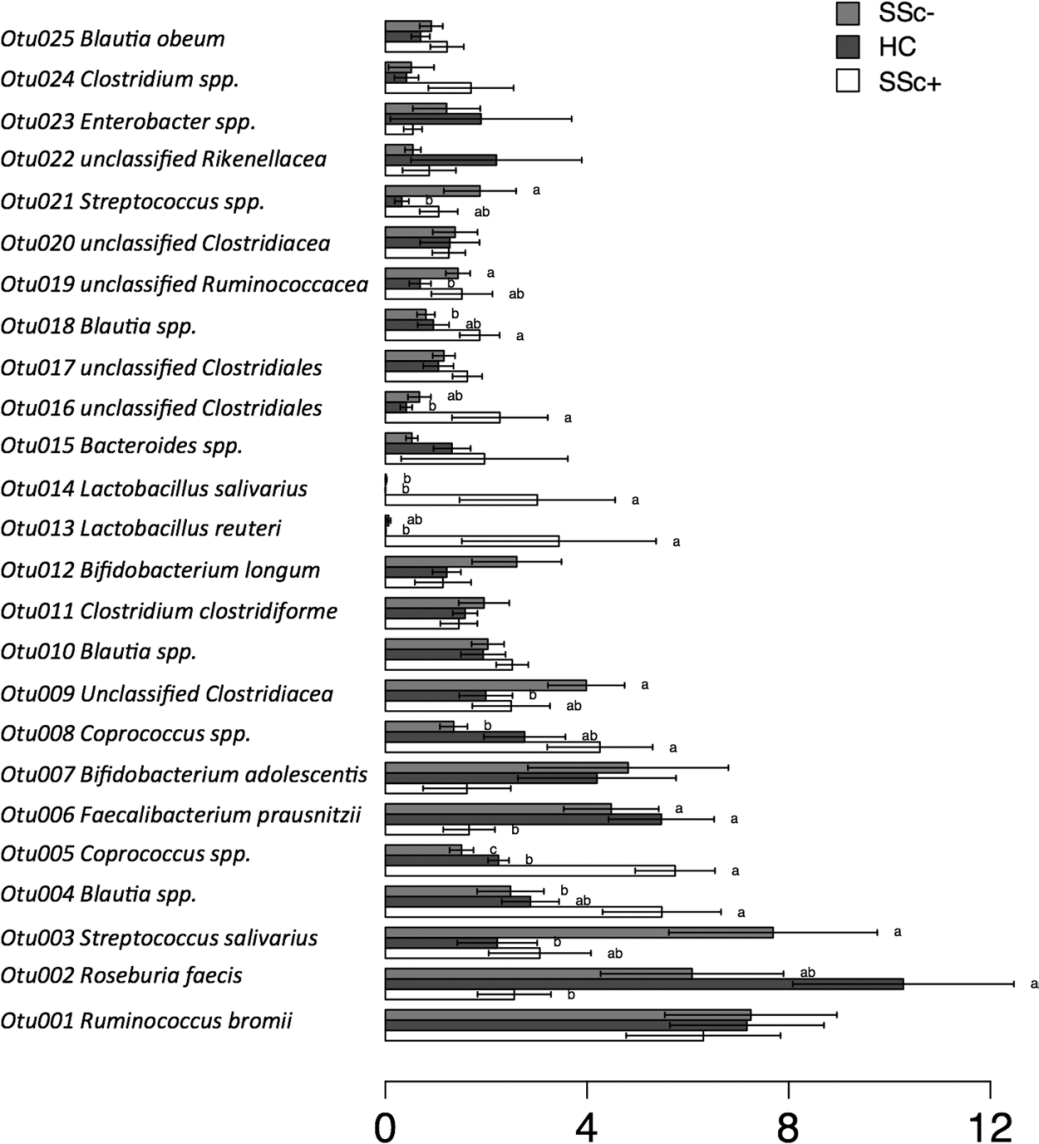
Differential relative abundance of specific OTUs across the three experimental groups. Metastats model was applied on the 25 most abundant OTUs with frequency >99%. The three experimental groups are indicated as SSc/GI+ (patients with established SSc and gastrointestinal symptoms), SSc/GI- (patients with SSc without gastrointestinal symptoms), and HC (healthy controls). Significant differences are highlighted by different minor letters (P < 0.05).

The hypothesis that specific OTUs can discriminate between healthy, SSc/GI+ and SSc/GI- subjects was further explored by Metastats, a technique to identify 16S-derived data (such as OTUs) whose relative presence is significantly different between treatments^21^. Figure 4 shows the relative presence and Metastats results for the 22 most abundant OTUs, which represented the 99% of the number of OTUs in the analyzed samples. For each OTU, the closest taxonomical classification is given, thus providing details about the taxonomical composition of the analyzed samples. The first nine OTUs had relative presence above 4% of the total bacterial community, and included *Ruminoccocus bromii*, *Roseburia faecis*, *Streptococcus salivarius* and *Faecalibacerium prausnitzii*. In accordance with the results of multivariate analyses, a number of these OTUs had significant differences between samples. Specifically, OTUs classified respectively as a *Coprococcus* spp. (OTU006), *Lactobacillus reuterii* (OTU011), *Bacteroidetes spp*. (OTU012), an unclassified *Lachnospiracea* (OTU0017) were more abundant in the SSc/GI+ subjects, whereas *Roseburia faecis* (OTU001) and *Faecalibacterium prausnitzii* (OTU004) were reduced. Interestingly, also a *Streptococcus salivarius* (OTU003), was significantly higher in the SSc/GI- group, while not showing differences between healthy and SSc/GI- subjects.

**Figure 5:**
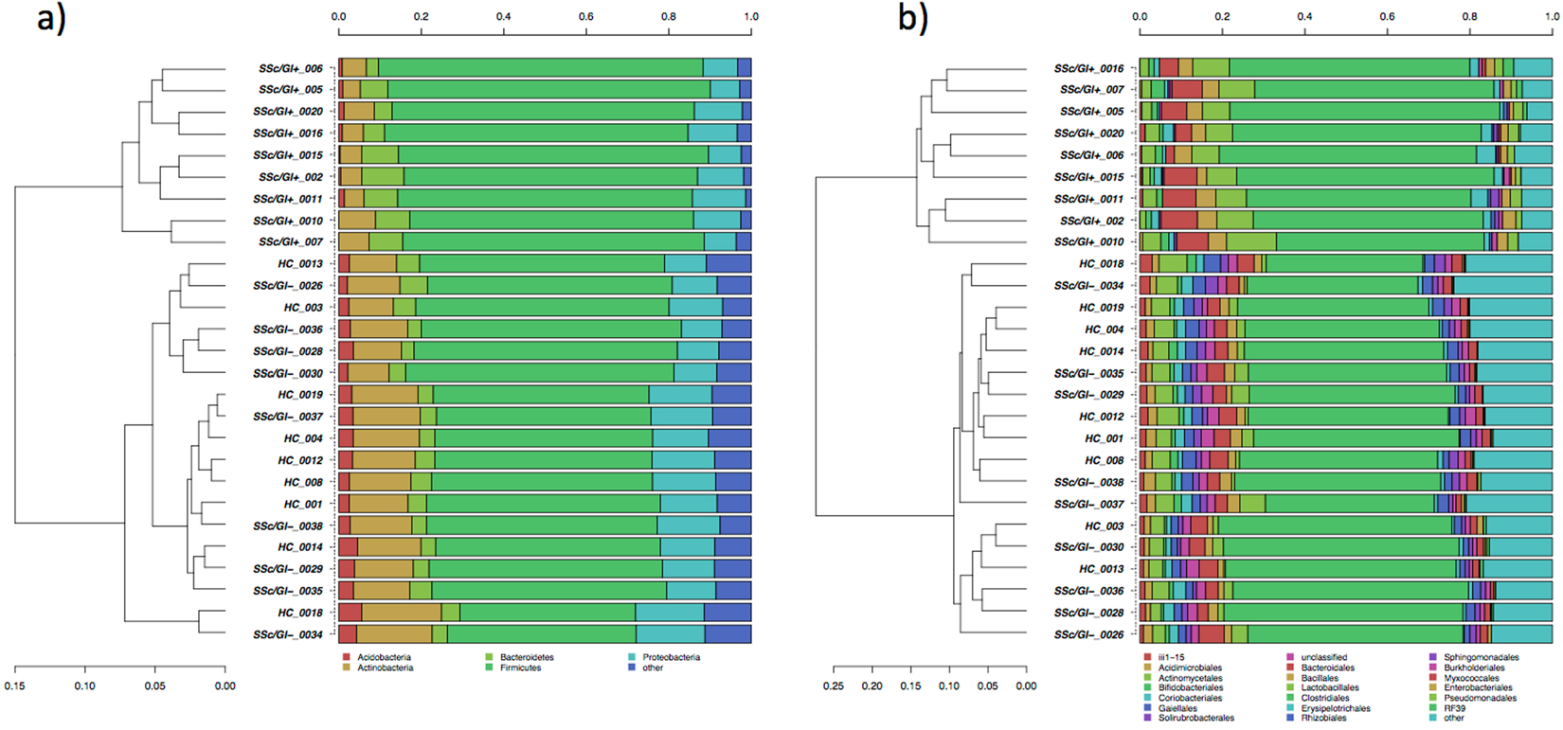
Hierarchical clustering of classified sequences at phylum (**a**) and order (**b**) classification level. The three experimental groups are indicated as SSc/GI+ (patients with established SSc and gastrointestinal symptoms), SSc/GI- (patients with SSc without gastrointestinal symptoms), and HC (healthy controls). Bars of different colors indicate he relative percentage of bacterial phyla (**a**) and orders (**b**) identified in each fecal sample. Only taxa participating with ≥5% in at least one sample are shown, while taxa with lower participations were added to the “other” sequence group. Similar samples were clustered using the average linkage algorithm.

Classification of OTUs at different taxonomic levels were analysed in order to assess the relative proportion of different bacterial taxa among samples, and to assess the clustering of individuals. When OTUs were classified at the phylum level, a dominance of *Firmicutes*, followed by *Actinobacteria* and *Bacteroidetes* was found as expected (Fig. 5a). All 9 SSc/GI+ subjects studied clustered separately already at the phylum level, namely because of a reduction in the relative proportions of *Actinobacteria* and *Bacteroidetes*. When the clustering analysis was repeated on sequences classified at the order level, a more detailed picture of the bacterial communities was obtained (Fig. 5b). All 9 SSc/GI+ subjects formed two separate clusters, and it was possible to find how the SSc/GI+ subjects generally had lower relative presence of Bifidobacteriales and a higher abundance of Clostridiales and Lactobacillales, in accordance with the significant increase of *L. salivarius* highlighted by the Metastats analysis on OTUs (Fig. 4).

**Figure 6:**
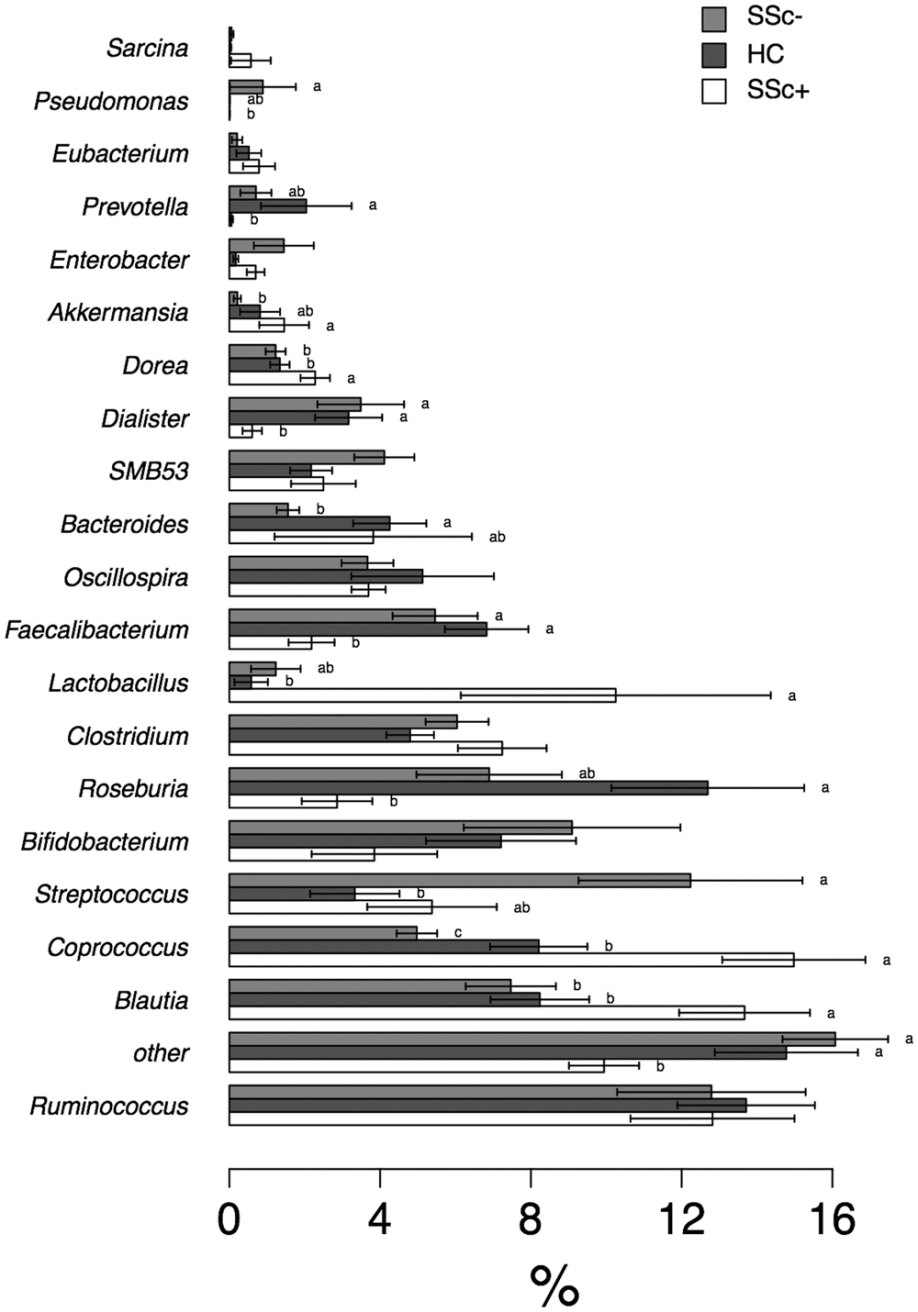
Differential relative abundance of specific genera across the three experimental groups. Metastats model was applied on the bacterial genera with a relative abundance of more than 5% in at least one sample. Genera with lower abundances were grouped in “other”. The three experimental groups are indicated as SSc/GI+ (patients with established SSc and gastrointestinal symptoms), SSc/GI- (patients with SSc without gastrointestinal symptoms), and HC (healthy controls). Significant differences are highlighted by different minor letters (P < 0.05).

The Metastats approach was applied also to assess any significant difference between treatments in the relative abundances of bacterial genera (Fig. 6). Fourteen genera in total were found to have a relative abundance ≥5% in at least one sample, with a number of significant differences between groups. In particular, SSc/GI+ subjects had higher relative abundances of *Blautia*, *Lactobacillus*, *Eubacterium*, *Bacteroides* and *Acinetobacter* while *Streptococcus*, *Ruminococcus* and *Roseburia* were significantly lower.

## Discussion

The results of the present study showed that bacterial diversity in terms of both richness and evenness varied significantly between healthy controls and SSc/GI+ patients, whereas no statistically significant differences were found between patients without gastrointestinal symptoms and healthy individuals. Interestingly, the fecal microbiota of SSc/GI+ patients had significantly lower richness but higher evenness than healthy controls, suggesting a decrease in the proportions of predominant bacterial types and/or an increase in the abundance of subdominant populations.

Beta-diversity analyses revealed that fecal bacterial communities of SSc/GI+ patients were characterized by a higher degree of inter-individual variability compared to both healthy and SSc/GI- subjects. This could reflect variable microbial ecology conditions in the gut of scleroderma patients with gastrointestinal disease associated with differences in host genotype and clinical phenotype. In this line of evidence, a much more variable microbiota was found to be associated with ileal Crohn’s disease, an inflammatory bowel disease (IBD) phenotype^22^. We therefore tried to find out those bacterial features that could discriminate the three groups of subjects under study and that were significantly associated with disease.

The majority of significant shifts found between healthy and the phenotypes of SSc/GI+ patients strongly involved bacterial taxa belonging to the Firmicutes order Clostridiales and in particular to the families Lachnospiraceae and Clostridiaceae. We observed a decrease of *Roseburia, Clostridium and Ruminococcus* at the genus level, and *R. faecis* and *F. prausnitzii* at the species level, in SSc/GI+ subjects compared with healthy people. These genera are dominant in the healthy human gut and are major producers of butyrate, which not only provides an important energy source for colonocytes, but also enhances intestinal epithelial barrier function and controls mucosal inflammation^23^. Recent studies have shown that genera such as *Roseburia*, *Faecalibacterium* and *Ruminococcus* are typically reduced in inflammatory disease states such as IBD^24–26^. *Roseburia* was also found to be lower in early-onset rheumatoid arthritis^27^ and *F. prausnitsii* in children with enthesitis-related arthritis^28^. These changes were associated with an increase of other taxa from the *Clostridium* XIVa cluster, namely the genus *Eubacterium*, and of some OTUs belonging to the genus *Coprococcus* and *Blautia*, although the latter did not reach statistical significance. In previous studies on immune-mediated diseases, *Coprococcus* was found to be inversely associated with psoriasis with or without arthritis (PsA)^29^ and the genera *Eubacterium* and *Coprococcus* to be generally reduced in patients with IBD^30,31^.

Surprisingly, an increase in *Lactobacillus* genus and in *L. reuteri* at the OTU level, was observed in SSc/GI+ patients. This finding is consistent with that of Volkmann *et al*.^16,17^ who found *Lactobacillus* to be significantly augmented in abundance in SSc subjects. Such result is unexpected since there is a large evidence gathered in human and animal studies showing the ability of strains from *L. reuteri* species to ameliorate gut inflammatory disease. Nevertheless, in a recent report, real-time PCR analysis of biopsy specimens of active CD patients showed that *Lactobacillus* group was increased in active IBD patients^32^. Contrary to what found by Volkmann *et al*.^16^ and in agreement with Andreasson *et al*.^33^, we did not observe any increase in *Bifidobacterium* spp. levels, rather we found a trend towards a decrease of this genus, and a significant decrease of *Bifidobacterium adolescentis* at the OTU level, in fecal samples from SSc/GI+ patients. However, it must be taken into account that differences in the observed bacterial variations may be linked to the different sample source, i.e. colonic mucosal lavage samples obtained during colonoscopy in the study of Volkmann and collaborators as opposed to fecal samples in our study. A further feature discriminating SSc/GI+ patients from healthy individuals were represented by an increase in the genus *Acinetobacter*; this finding is consistent with the earlier work of Volkmann *et al*.^17^ who found *Acinetobacter* in greater abundance in Norwegian SSc patients compared with controls*. Acinetobacter* is mainly found on human skin and in nasal cavities, although it has also been detected in the human gut. It has been suggested that multiple sclerosis could be triggered by *Acinetobacter* infection within the nasal sinuses^34^ and *Acinetobacter* spp. isolated from the skin microbiome of individuals with STAT1/STAT3 defects inhibited the capacity of human primary leukocytes to produce proinflammatory cytokines^35^.

In this study, the microbiota of SSc patients without gastrointestinal disease was investigated in order to check out if microbial features representative of an early marker of dysbiosis could be found. As expected, these subjects showed a composition more similar to that of healthy subjects than to that of patients with SSc/GI+ in terms of both diversity and taxonomical composition. Surprisingly, they showed some perturbations in the relative abundances of bacterial populations not occurring in SSc/GI+ patients or even displaying an opposite trend. SSc/GI- subjects had higher percentages of *Streptococcus* spp. (and specifically of *S. salivarius*) compared to healthy individuals. This result is interesting as it has been recently demonstrated that *S. salivarius* strains exert regulatory effects on the NF-κB pathway in human intestinal epithelial cells and may thus play a protective role in gut inflammatory homeostasis^36,37^. When compared to SSc/GI+ people, SSc/GI- patients were specifically characterized by significant lower levels of *Blautia*, *Dorea and Bacteroides*. Notably, *Dorea* is a major gas-producing bacterium in the human gut^38^ and it is tempting to speculate that an increased production of gas could probably account for the abdominal bloating experienced by SSc patients with gastrointestinal tract involvement. *Blautia* is able to utilize hydrogen and carbon dioxide to form acetate, and its increase in SSc/GI+ patients might be tightly linked to that of *Dorea*. It has been suggested that the increased levels of *Blautia* spp. as well as *Dorea* spp. observed in subjects with irritable bowel syndrome (IBS) could reflect the adaptation of the former to a larger amount of gases produced by the latter^39^.

Overall, our results confirm those of Volkmann and collaborators^16,17^ in that the gut community of scleroderma patients is characterized by distinct shifts in microbiota composition and warrant further investigation to understand whether these bacterial alterations contribute to the disease or merely reflect secondary changes caused by intestinal inflammation and/or other disease-associated conditions. Compared to other diseases in which the dysbiosis of gut microbiota raises the issue of causality between microbioma and clinical states, systemic sclerosis is unique in that gut involvement is one of the features of the disease affecting a large proportion of patients^3,4^, and contributing to dysbiosis^33,40^ in which small intestinal bacterial overgrowth, which has been detected in up to 43.1% of SSc patients^40^, may as well concur. Thus, in SSc/GI+ patients it is unclear whether dysbiosis of gut microbiota reflects intestinal involvement induced by the mechanism(s) involved in the pathogenesis of SSc (e.g., vascular or immunologic mechanisms), or is the driving force of the pathogenesis of all the abnormalities of the disease as well as of the gut, as suggested by the “common ground” hypothesis according to which the dysbiotic microbial communities may promote both local and systemic alterations^41^. The issue is further complicated by the evidence that immunological mechanisms are operating in SSc as a whole as well as in gastro-intestinal disease^42,43^ and, hence, modulated by intestinal microbiota the role of which in the maturation and education of the host immune response is well established^44^. On the other hand, the differences in the levels of several taxa between SSc/GI- subjects and healthy individuals did not quite coincide with those observed in SSc/GI+ patients. These results raise a very important question, i.e. if the alterations occurring in SSc/GI- gut microbiota might reflect a steady-state situation or a transitional state eventually evolving towards more severe disease outcomes.

Some aspects of the present study, however, deserve few further comments. First, even if it could be assumed that all or some of the SSc/GI- could have had asymptomatic gastrointestinal disease, it can be speculated that GI tract involvement was not so relevant to cause symptoms that required medical attention or induce abnormalities of the diagnostic tests. Thus, the differences in gut microbiota composition between SSc/GI- and SSc/GI+ patients are still noteworthy. Second, SSc/GI+ patients had evidence of a more severe disease compared to SSc/GI- subjects. As stated above, further studies are warranted to clarify whether the microbial dysbiosis is a consequence or a cause of all or some disease manifestations. Third, stool consistency, F- calprotectin, and fecal butyrate, as well other biochemical parameters that could help understand the pathogenesis of SSc gastrointestinal disease, were not evaluated. We also want to point out the high female ratio of this study correctly reflects the disease distribution; controls were selected accordingly. Lastly, since GI tract involvement in SSc patients may be heterogeneous, we should be cautious to link the composition of the gut microbiota we detected to all the features of GI tract disease of SSc patients. In fact, we have only evaluated fecal microbiota, which does not necessarily reflect what occurs in other parts of the GI tract.

Future studies dealing with longitudinal studies of the fecal microbioma in these patients and their clinical status, complemented by mechanistic and functional investigations, including measurements of short chain fatty acids (acetate, butyrate, and propionate) and F- calprotectin in faeces may help clarify the role of microbial communities in the pathogenesis of systemic sclerosis and associated gastrointestinal manifestations.

## Materials and Methods

### Patients

The study involved 18 consecutive SSc patients fulfilling the diagnosis of systemic sclerosis according to the ACR/EULAR 2013 criteria^45,46^. All controls and patients were evaluated with the UCLA SCTC GIT 2.0 questionare. All patients underwent glucose and lactulose H_2_/CH_4_ breath tests for small intestinal bacterial overgrowth, TC scan of the chest for esophageal dilatation, modified barium swallow for esophageal dysmotility.^13^C octanoic acid breath test was performed in 4 patients all of whom tested positive.

Nine patients had symptoms assessed with the UCLA SCTC GIT 2.0 instrument^47^, and evidence of gastrointestinal involvement (SSc/GI+). The other 9 patients did not report symptoms of gastrointestinal involvement and had no evidence of gastrointestinal disease (SSc/GI-) after the following investigations: modified barium swall for esophageal motility, glucose H2/CH4 breath test for small intestinal bacterial overgrowth and lactulose breath test for gastric emptying.

Inclusion criteria were: SSc, as defined above, age 18 to 80 years, and ability to give informed consent. Pregnancy had to be ruled out before the beginning of the study. Exclusion criterion was the presence of an infectious diseases at study entry. The study participants received no antibiotic treatment, probiotics, prebiotics, steroids, immunosuppressive drugs or any other medical treatment influencing intestinal microbiota during the 3 months before the start of the study, and proton pump inhibitors 3 weeks before the tests. None of the patients were on steroids or immunosuppressive drugs at the time of enrollment, and if necessary, treatment was postponed. The control group (HC) included 9 age and sex matched healthy subjects. They were selected according to the following inclusion criteria: good health, no clinical history of autoimmune diseases, free from any medical therapy that could interact with the gut microbiota. Healthy controls, SSc/GI+ and SSc/GI- groups had the same dietary and lifestyle habits assessed with the Healthy Lifestyle and Personal Control Questionare^48^.

The protocol, patient information sheet and consent form were prepared as in two previous pharmacological studies of our group (n# 2007-00532268 and # 2013-004596-12) approved by the local Ethics Committee of Marche Region, Italy (27/9/2007 and 282/2/2014, respectively). The study was conducted in accordance with the Declaration of Helsinki in its fifth edition (2000). Written informed consent was obtained from all patients.

Stool samples were collected at home and delivered to the storage area for frozen storage at −80 °C within one hour.

### DNA extraction

Genomic DNA was extracted from 200 mg of faecal samples by using the Fast DNA™ SPIN Kit for Soil (MP Biomedicals, Santa Ana, CA) and the FastPrep®-24 Instrument following the manufacturer’s instructions. DNA integrity was checked by agarose gel electrophoresis and DNA quantity measured by Qubit HS dsDNA fluorescence assay (Life Technologies, Carlsbad, CA, USA).

### 16S rRNA gene amplification and high-throughput sequencing in Illumina

PCR amplicons covering the V3-V4 regions of the 16S rRNA were analysed in Illumina MiSeq with V3 chemistry in 300 bp paired-reads mode. PCR reactions were performed in 25 μL containing 12.5 μL of Phusion Flash High-Fidelity Master Mix (Thermo Fisher Scientific, Inc., Waltham, MA, USA), the primer pairs 343 F (5′-TACGGRAGGCAGCAG-3′) and 802 R (5′-TACNVGGGTWTCTAATCC-3′) in 0.5 μM each, 1 ng of DNA template (as determined with QuBit measurements) and PCR grade water. In order to analyse several amplicon samples simultaneously in the same sequencing run, a multiplexing strategy was employed by adding a 9 nucleic acids extension to the 5′ end of the forward primer, where the first seven bases served as a tag to identify to each sample, and the following two bases were a linker designed not to match bacterial sequences in the same position according to RDP entries, as previously described^49^. To reduce possible biases related to the primer extension, the two step-PCR approach described in Berry *et al*.^50^ was adopted, with a first PCR step of 25 cycles using untagged primers, and a final step of 8 cycles with tagged primers and 1 μL of first step products used as template. The PCR conditions were the same in both steps, and consisted in 30″ of denaturation (94 °C), 30″ of primers annealing (50 °C) and 30″ of primers elongation (72 °C), followed by a final elongation step (72 °C) of 10 minutes. The final PCR products of each sample were checked on 1.5% agarose gel and pooled in equimolar amounts according to QuBit measurements. The final pool was then cleaned with the SPRI (Solid Phase Reverse Immobilization Method) using the Agencourt Agencourt^®^ AMPure^®^ XP kit (Beckman Coulter, Italy, Milano). The pool was finally sequenced by Fasteris Company (Geneva, Switzerland) with a MiSeq Illumina instrument (Illumina Inc, San Diego, CA) operating with V3 chemistry and producing 300 bp paired-reads.

### Sequences data preparation, bioinformatics and statistical analyses

High-throughput sequencing data filtering, multiplexing and preparation for concomitant statistical analyses were carried out as previously detailed^51,52^. In summary, paired-reads were assembled to reconstruct the full V3-V4 amplicons with the “pandaseq” script^53^ allowing a maximum of 2 mismatches and at least 30 bp of overlap between the read pairs. Samples demultiplexing was then carried out with the Fastx-toolkit (http://hannonlab.cshl.edu/fastx_toolkit/).

Mothur v.1.32.1^54^ was applied in order to remove sequences with large homopolymers (≥10), sequences that did not align within the targeted V3-V4 region, chimeric sequences^55^ and sequences that were not classified as bacterial after alignment against the Mothur version of the RDP training data set. The resulting high-quality sequences were analysed with Mothur and R^56^ following the operational taxonomic unit (OTU) approach. Sequences were first aligned against the SILVA reference aligned database for bacteria^18^ using the NAST algorithm and a kmer approach^57,58^ and then clustered at the 3% distance using the average linkage algorithm. OTUs were classified into taxa by alignment against the Greengenes database^59^.

Statistical analyses on OTU matrixes were performed in Mothur and R, and included hierarchical clustering with the average linkage algorithm at different taxonomic levels, principal component analysis (PCA) and canonical correspondence analyses (CCA). Metastats was applied to identify OTUs and genera that were significantly different between treatments^23^.
